# The U.S. Needs a National Human Health Observing System

**DOI:** 10.3389/fpubh.2021.705597

**Published:** 2021-09-06

**Authors:** Paul A. Sandifer, Burton H. Singer, Rita R. Colwell

**Affiliations:** ^1^Center for Coastal Environmental and Human Health, School of Sciences and Mathematics, College of Charleston, Charleston, SC, United States; ^2^Emerging Pathogens Institute, University of Florida, Gainesville, FL, United States; ^3^Johns Hopkins University Bloomberg School of Public Health, University of Maryland Institute for Advanced Computer Studies, University of Maryland College Park, Baltimore, MD, United States

**Keywords:** health observing system, health policy, COVID-19, disasters, cohort studies, health surveillance, Gulf of Mexico

## Abstract

The COVID-19 pandemic and increasing frequency and severity of environmental disasters reveal an urgent need for a robust health observing/surveillance system. With the possible exception of Brazil, we know of no such comprehensive health observing capacity. The US should create a national system of linked regionally-based health monitoring systems similar to those for weather, ocean conditions, and climate. Like those for weather, the health observing system should operate continuously, collecting mental, physical, and community health data before, during, and after events. The system should include existing cross-sectional health data surveys, along with significant new investment in regional longitudinal cohort studies. The recently described framework for a Gulf of Mexico Community Health Observing System is suggested as a potential model for development of a nation-wide system.

## Introduction

Our objectives in this short communication are to illustrate the overarching need for a national human health observing system, provide an example of what such a system might encompass, and outline two steps, one near-term and one medium- to long-term, that could be taken toward its development.

More than any previous disaster of the twenty or twenty first centuries, the COVID-19 pandemic, and the increasing likelihood of more emerging diseases in the future, have highlighted an urgent, overarching need for comprehensive health surveillance at local to national scales (1–4). The conditions in the US earlier in the pandemic and the current crises in Brazil (5) and India (6) are stark examples of the need. This pandemic, like numerous previous ones, is likely the result of a zoonotic disease, a cross-over from unidentified animals (most probably bats) to humans (7, 8). It is far from the last pandemic likely to occur, based on the history of poorly regulated legal and unrestrained illegal wildlife trade that regularly results in human exposure to potentially deadly wildlife diseases (9–12). That, in addition to the exponential global growth in human travel and material transport (13, 14), makes the probability very high. The current pandemic is causing high mortality levels and serious physical and mental health impacts in the populations of countries around the world. The adverse health effects are more severe for vulnerable groups, namely the aged, those with underlying health conditions, the disabled, people of minority ethnicity (especially native Americans, African Americans, and Hispanics), people living or working in communal residences such as nursing homes, the military, and prisons, and others. In addition, there is a very serious need to track and alleviate the stresses associated with illness anxiety, management of stay-at-home work and education, social distancing, business closures and job losses (15). These factors become more complicated in unrelated but simultaneously occurring life- and livelihood threatening circumstances, in particular the mandatory evacuations and destruction associated with disasters, notably hurricanes, tropical storms, tornados, floods, and wildfires that occurred at unprecedented levels in 2020 and portend to occur more frequently and severely in the future (16, 17). Dealing with potential health impacts, and tracking their effects and treatment over time, of even one such disaster is fraught with difficulty. The possibility of having to deal with multiple and perhaps simultaneously occurring disasters amidst an ongoing pandemic is even more daunting. Of high concern is the likely occurrence of excess morbidity and mortality, i.e., not directly tied to the pandemic but resulting from delayed and/or postponed treatment and preventive medical care for other conditions unrelated to the disaster as occurred following Hurricanes Katrina and Maria (18–20).

## Policy Options and Implications

One recommendation suggested by Gottleib et al. (2) to the CDC (Centers for Disease Control and Prevention) was to “convene an intergovernmental task force, with outside experts as needed and input from states and the health care community, to develop and support a new national surveillance system and data infrastructure for tracking and analyzing COVID-19.” Similarly, among recommendations for improving pandemic preparedness and resilience in the U.S., Daszak et al. (21) emphasized strengthening the nation's public health systems and establishment of an independent commission led by the National Academies of Science, Engineering, and Medicine (NASEM) to review the US pandemic response and develop strategies for the future. We agree with these suggestions, but believe they may be overly focused on the current health emergency, namely COVID-19, and insufficiently broad to encompass not only future disease epidemics, but also the aggregated health effects from multiple types of disasters and associated collateral social and economic damage. Acknowledging obstacles that derive from the balkanized US health care system, we recommend establishing regionally-based, interlinked longitudinal cohort studies that will provide early warning and tracking of pandemic disease and other health issues at a country-wide scale. Specifically, we highlight the need for a health observing system that combines both cross-sectional and individual patient data with information from specifically designed longitudinal cohort studies. Unfortunately, no comprehensive health surveillance system that is representative of the population, inclusive of the vulnerable, longitudinal and continuous, and incorporates information from ongoing cross-sectional surveys and other studies, presently exists in the U.S. Regrettably, the national health system in Brazil is being degraded, but it provides examples of what could be done (22). In the U.S., the need for prospective cohort studies for COVID-19 was identified relatively early in the pandemic (4), and repurposing some ongoing longitudinal cohort studies is in process (23). In addition, a few relatively short-term prospective and retrospective cohort studies targeted to COVID-19 in specific localities are underway (24, 25), and the National Institutes of Health (NIH) is supporting a National COVID Cohort Collaborative. The latter is to serve as “a secure portal for patient-level COVID-19 clinical data” (26), and it may be possible to utilize the All of Us national cohort study (https://www.nih.gov/news-events/news-releases/all-us-research-program-launches-covid-19-research-initiatives). Unfortunately, the sampling being done by the All of Us study lacks a statistically-based approach, thus limiting the epidemiological utility (27). Thus, a comprehensive, prospective cohort-based system at the regional to the national scale is not now available. Lack of a health surveillance capacity hampers all efforts, from monitoring numbers of infections, to effects of the disease and treatment, long-term and unforeseen consequences, and even the ability to determine when/if a recovery has begun and at what rate of progress. Of notable importance is the need to monitor vaccine demand, distribution and uptake in the event it becomes necessary to administer annual vaccinations basis against additional variants of SARS-CoV-2 virus, the causative agent of COVID-19.

Prior to onset of the COVID-19 pandemic, with its massive health, economic, and social impact, a diverse team of scientists and health professionals with expertise in disaster response, public health, medicine, epidemiology, observing systems, and other relevant areas of environmental health science had focused on health information needed for the disaster-prone Gulf of Mexico region. In addition to a series of environmental disasters, including Hurricane Katrina, the Deepwater Horizon oil spill, and six land-falling tropical cyclones in 2020 alone, Gulf residents continue to suffer long-term health, socio-economic, and educational disparities (28–30). Climate change, subsidence, development pressures, harmful algal blooms and other ocean-associated health threats, and the COVID-19 pandemic are examples of recent calamities that are expected to increase in occurrence and severity. Recent research revealed a critical lack of availability of baseline health data captured before, during, and continuing long after a disasters. It was concluded that ongoing health monitoring is essential to reduce and manage health impacts of future disasters. In this regard, (31) highlighted the crucial importance of acquiring and using baseline scientific information in any response to a societal crisis. In summary, to address the need outlined in this communication the Research Board of the Gulf of Mexico Research Initiative commissioned a study to develop a framework for a health observing system. An interdisciplinary team undertook this assignment under the leadership of P. Sandifer and B. Singer. The work was accomplished through two expert workshops, consultations with knowledgeable individuals, review of an extensive body of literature and ongoing health surveys, and iterative design and writing efforts, and was informed by existing environmental observing systems (32, 33).

**Figure 1:**
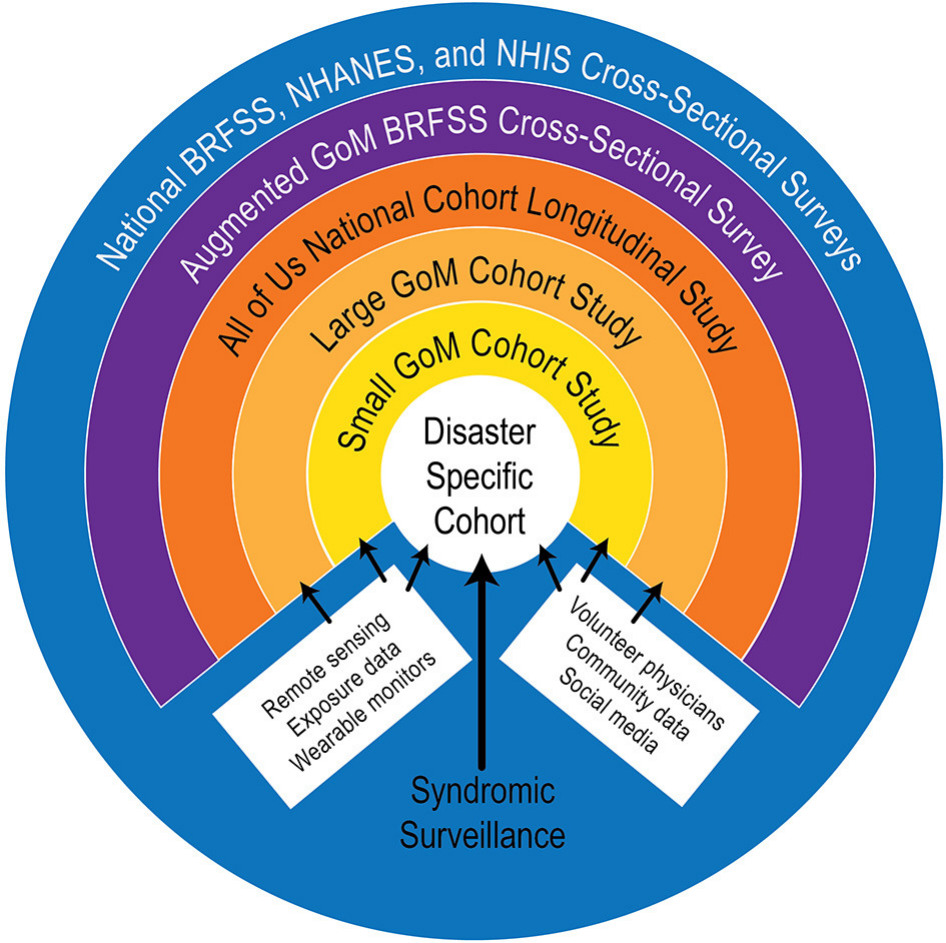
Schematic framework for a Gulf of Mexico Community Health Observing System [from Sandifer et al. (33)].

The resulting design builds on existing national health surveys and studies (32, 33). Most importantly, it has added several longitudinal cohort studies focused explicitly on vulnerable coastal areas and includes specific disaster-responsive components, representative and targeted population sampling, and self-reported and clinically-derived demographic, psychosocial, and physical health data. The health observing system framework, illustrated conceptually in Figure 1, takes advantage of available and developing technologies such as electronic health records, wearable health devices, remote sensing, advances in genomic and other omic sciences and embraces community-based participatory research approach as an integral operating principle. Importantly, the proposed Gulf health observing system incorporates measures of chronic and cumulative stress that utilize biomarkers to identify allostatic load, an index of toxic stress. Toxic stress is known to be a pervasive problem associated with disasters and other traumatic events and it often leads to long-term psychosocial and physiological pathologies (33–39). In fact, harmful effects of massive stress caused by childhood exposure to the Holocaust was recorded by Holocaust survivors more than 70 years after the fact (39). Peters et al. (40) posited that pre-disease psychosocial assessments of chronic stress could help guide COVID-19 treatments.

The Gulf of Mexico Health Observing System (GoM CHOS) framework design is based, in part, on the atmospheric and oceanic observing systems that underlie modern weather, climate, and marine forecasting capabilities, upon which hundreds of millions to billions of people depend daily. These environmental observing systems enable society to mitigate effects of extreme weather events and resulting costly health and socio-economic impacts at local to global scales. Using the GoM CHOS as a model for pilot studies and building out to a regional framework, as employed by the US Integrated Ocean Observing System (IOOS) (https://ioos.noaa.gov/regions/), could provide a nation-wide health observing system of unparalleled capacity.

As noted in Sandifer et al. (32, 33), risks to participants in a health observing system are expected to be relatively minor and include lapses in data security, anxiety or discomfort in providing personal information or associated with clinical visits and/or biospecimen collection, and programmatic issues such as continuity, funding, and management. Significant participant benefits include regular medical checkups, continued health monitoring over time, increased personal and community resilience, and enhanced personal preparedness to deal with health consequences of future disasters and other traumatic events. At a larger scale, information from health observing systems can lead to development of public health strategies that help prevent or mitigate disaster-associated health hazards and propel innovation in medical research and practice. Other larger scale advantages deriving from a nation-wide health observing system are presented in the conclusions section of this communication.

Establishment of a nation-wide structure of linked regional health observing systems patterned after the GoM CHOS framework or some other models that include both cross-sectional and representative longitudinal cohort components can be a major step toward a national health observing/surveillance capability. Connecting regional health observing units to academic health centers, the private sector, and data management centers will increase the reach and utility of the health observing system significantly. Clinical data collection components can include testing for SARS-CoV-2 or other potential diseases of known concern, and new sampling and diagnostic technologies for emerging diseases and health issues can be incorporated in existing longitudinal cohorts. Ongoing longitudinal cohorts will provide mechanisms for tracking prevalence and health effects over time, as well as association or interaction with other health factors including psychosocial and physiological stress, effectiveness of vaccines, and other preventative methods, and treatments. A national health observing system can provide data needed by public health officials, clinicians, researchers, and the public to monitor progress in managing health effects of future pandemics, environmental disasters, and economic disruptions.

## Actionable Recommendations

We recommend the following short- and medium-to long-term actions:

1) Short-term: As a first step, the Federal Government or Congress should authorize a Federal agency (or group of agencies) to undertake a health observing system pilot program and it could be in the Gulf of Mexico region to take advantage of the Gulf of Mexico Community Health Observing System framework. Wherever and however conducted, the purpose of the pilot would be “to learn by doing,” to establish a strong base of information for designing and building a national network of regional health observatories. Although cost estimates were not a part of the original study, a rough “guestimate” of funding required for a Gulf region health observing system is ca. $10 M/year. Better estimates will depend on implementation decisions (33). Almost certainly, a robust pilot project could be undertaken with financial support at this level, although a system at national scale would require considerably more funding. In this light, we note that in 2016, before the pandemic, the U.S. Congress authorized funding of $1.5 B over 10 years for the NIH All of Us study (https://allofus.nih.gov/news-events/press-kit/all-us-research-program-backgrounder).
2) Medium to long-term: The Federal Government, with leadership of the CDC, NIH, and NSF and participation of the NASEM, should establish a Commission or Committee to make recommendations to the President and the Congress for creation and long-term funding of a national system of regional health observing systems focused on pandemics and disasters. The Commission or Committee should comprise public health and biomedical experts with relevant expertise from Federal Government health agencies, State Health Departments, public health and medical experts from academic institutions, public and private health care providers, computational data specialists, and others as may be required.

## Conclusions

The COVID-19 pandemic and the increasing frequency and severity of environmental disasters make obvious the urgent need for a robustly operational health observing system for the U.S. A comprehensive health observing capacity does not exist and should be developed. A regionally based, nationally linked system, as outlined in this communication, can serve to gather the health information, including mental, physical and community health data, in a representative sampling of the nation's population, including the susceptible or vulnerable, with respect to effects of pandemics and other disasters. The database would be available to public health officials, medical practitioners, disaster preparedness planners, biomedical researchers, and others to improve health preparedness and response. Multiple health observing systems would create comparable, ongoing health data streams that could improve targeting of investments for disaster preparedness, response, and prevention, there by reducing future expenditures for long-term health care related to disasters. Also, such systems could provide earlier and stronger warnings of impending epidemics, as well as tracking of possible long-term effects and contacts, allowing more targeted and possibly less severe responses, which would reduce economic and other societal impacts. Although aspirational at present, a national health observing system is feasible. Considering the high annual costs of disasters and the massive cost of the COVID-19 pandemic, not taking action to improve health monitoring will be far more expensive.

## Author Contributions

PS, BS, and RC were responsible for concept formulation. PS had primary funding and writing responsibilities. All authors contributed ideas, reviewed, and approved the manuscript.

## Author Disclaimer

The content of this paper is solely the responsibility of the authors and does not necessarily represent the official views of the Gulf of Mexico Alliance, the Gulf of Mexico Research Initiative, the National Institute of Environmental Health Sciences, the College of Charleston the National Science Foundation, or the University of Maryland College Park.

## Conflict of Interest

The authors declare that the research was conducted in the absence of any commercial or financial relationships that could be construed as a potential conflict of interest.

## Publisher's Note

All claims expressed in this article are solely those of the authors and do not necessarily represent those of their affiliated organizations, or those of the publisher, the editors and the reviewers. Any product that may be evaluated in this article, or claim that may be made by its manufacturer, is not guaranteed or endorsed by the publisher.
